# Genetics of Color Polymorphism in the Pea Aphid, *Acyrthosiphon pisum*

**DOI:** 10.1673/031.010.9501

**Published:** 2010-07-08

**Authors:** Marina C. Caillaud, John E. Losey

**Affiliations:** ^1^ Department of Biology, 165 CNS, Ithaca College, Ithaca, NY 14850; ^2^ Department of Entomology, Cornell University, Ithaca, NY 14853, USA

**Keywords:** autosomal locus, body color, Mendelian genetics

## Abstract

The genetic basis of color polymorphism is explored in the pea aphid, *Acyrthosiphon pisum* (Harris) (Homoptera: Sternorrhyncha), in which two color morphs have been described (pink or green). Laboratory crosses and a Mendelian genetic analysis reveal that color polymorphism in pea aphids is determined by a single biallelic locus, which we name *colorama*, with alleles *P* and *p*, pink being dominant to green. The putative genotypes are *Pp* or *PP* for pink morphs, and *pp* for green morphs. This locus is shown to be autosomal. Last, there was no evidence of influence of the direction of the cross on color inheritance, thus showing that cytoplasmic effects and/or maternally-inherited symbionts play no role in the inheritance of color polymorphism in pea aphids. The existence of a simple genetic determinism for color polymorphism in a system in which genetic investigation is possible may facilitate investigations on the physiological and molecular mechanisms of genetically-based color morph variation, and the establishment of a link between this locus and fitness in a range of ecological conditions.

## Introduction

Color polymorphisms provide some of the best characterized examples of functionally and ecologically important polymorphisms. In many animal taxa, color polymorphism differentially affects the fitness of individuals in a species. Some color morphs may, for instance, be more cryptic than others and therefore harder for predators to locate visually. Examples of differential crypsis and risk of predation among color morphs have been reported in vertebrates as well as in invertebrates (review in Majerus 1998; Hoekstra and Nachman 2003; Petranka et al. 1998). One of the most famous cases involved the color morphs of the peppered moth *Biston betularia* (review in Majerus 1998). Alternatively, a color polymorphism may enhance an individual's chance of reproduction by affecting thermoregulation (Foreman 1999; Forsman et al. 2002; de-Jong and Brackenfield 1998).

Many fitness-related traits of ecological relevance are quantitative and unlikely to have a simple genetic basis. This makes the establishment of a connection between genotype and phenotype difficult and considerably hinders understanding of genetic variation and its evolutionary impacts (for recent exceptions, see Daborn et al. 2002; Dobley et al. 1997). In contrast to most phenotypic traits of ecological relevance, several cases of color polymorphism have a simple genetic basis. Early reported examples include the single-locus polymorphism for color pattern in the snow goose *Lampropeltis caerulescens* (Pough 1951) and the ladybird *Harmonia axyridis* (Tan 1946). More recently, color morph variation in several species of damselflies was shown to be controlled by one three-allele autosomal locus (Cordero 1990; Andres and Cordero 1999). Thus color polymorphisms may offer a situation in which finding genes that underlie adaptation can be particularly successful. This would in turn allow for the investigation of the type of genetic changes associated with adaptation (are changes in the coding or in the regulatory region of the genes involved?) and to examine the relationship between the type of selection pressure (abiotic versus predatory, for instance) and the type of genetic change. Several studies have shown how biotic and abiotic factors can exert stabilizing selection on color patterns in animals (Losey et al. 1997; Mallet and Barton 1989).

Among animal species, some of the widest ranges of color morphs within species and some of the most intensely studied polymorphic systems are found among insects (Kettlewell 1973 and several subsequent papers). Within the insects, aphids are an attractive family for study. First, color polymorphism is quite common (Dixon 1985; Weber 1985; Tomiuk et al. 1990; review in Lambers 1966). Second, color variation can exist between genotypes and even between asexually-produced forms of a single genotype. Third, aphids are often susceptible to an array of natural enemies that may be affected by variations in prey color, thus color variation in aphids is likely to be adaptive. The extent of color variation may be limited, as in the pea aphid *Acyrthosiphon pisum* (Harris) (Homoptera: Sternorrhyncha) which displays pink and green forms (Markula 1963), or extensive, as in the grain aphid *Sitobion avenae* which displays many color forms including green, brown, pink and chestnut (Weber 1985). The mechanisms responsible for the expression of color include, in different species, environmental (photoperiod and temperature), nutritional (plant and type of diet in general), bacterial symbionts, infection by pathogens, genetic factors, or a combination of one or more of these (Lambers 1966).

The exact nature of the impact of color polymorphisms on aphid fitness is usually unknown. However, in the pea aphid, *A. pisum,* color variation (pink versus green) was clearly shown to affect the susceptibility of individuals to predators and parasitoids (Losey et al. 1997). Green aphid morphs suffer higher rates of parasitism than pink morphs, while pink morphs were more prone to predation than green morphs. Moreover, color polymorphism in pea aphids may be under “bottom up” selective pressures from their host plants in addition to the “top-down” selective pressures from predators and parasitoids demonstrated in Losey et al. (1997). Although Losey and Eubanks (2000) found no significant difference in the ability of pink and green morph collected from forage crops to survive on various vegetable host plants, Kugler and Ratcliffe (1983) found pink and green morphs differed in their ability to utilize alfalfa hybrids that had been bred for resistance to aphids. Also, significant differences in color morph frequency were found in French fields of *Pisum sativum* (pea), *Medicago sativa* (alfalfa), and *Trifolium pratense* (red clover). In a sample of almost 1,000 individuals collected on those three plants, pink morphs represented 99.5% of the pea aphid populations on pea, but only 79% and 66% of the population on alfalfa and red clover, respectively (Simon et al. 2003). Last, pink aphids tended to drop more easily from a plant and to produce more winged offspring after disturbance than green morphs (Braendle and Weisser 2001;Weisser and Braendle 2001). In sum, pea aphid color polymorphism may be under a complex set of selective pressures from both higher and lower trophic levels, and appears to be associated with behavioral differences as well as host-plant preferences.

The mechanisms of color variation in aphids were first studied by Müller (1962, 1979, 1987). His general conclusion was that genetically-based variation in body color in a range of aphid species was the result of a single two-allele locus, one allele being dominant to the other. For instance, in the species *Aphis fabae cirsiiacanthoides,* he allowed a black morph to breed with a yellow morph, which gave an all-black F1. Crosses of these gave 5 black and 1 yellow F2 progeny (Müller 1962). Muller then concluded that color morph in *A. fabae cirsiiacanthoides* was influenced by one gene, black being dominant to yellow. However, little information on the details of his experimental methods was given. The size of the F1 and F2 progeny was very small (when this information was provided). Overall, little genetic data was given to support his conclusions about the genetic basis of color morph in the several aphid species investigated. In the pea aphid, efficient and reliable methods for performing controlled crosses aphids were developed by Via (1992) thus making possible studies that directly address the Mendelian genetics of several phenotypic traits of ecological and evolutionary significance aphids. For this paper, a modified version of these methods (Caillaud et al. 2002) was used and controlled crosses between green and pink morphs of the pea aphid were performed to explore the genetic basis of color polymorphism in this species. The genetic model for inheritance of color polymorphism in aphids proposed by Müller (1987) was tested, as well as whether this single biallelic locus is X-linked and whether cytoplasmic/symbiotic factors play a role in color inheritance.

## Materials and Methods

*A. pisum* are non-host-alternating cyclical parthenogens with a single sexual generation in the fall and many successive parthenogenetic generations from early spring to late fall. In the fall, a combination of cold temperatures and decreased photoperiods leads to the differentiation of unusual parthenogenetic females (sexuparae) capable of giving birth to sexual forms (sexual females and males) that will mate and produce cold-tolerant eggs. Sex determination is of the XX/X0 (female/male) type. Males are generated by an unusual oocyte division or “mini-meiosis” in which only one of the two X chromosomes carried by the sexuparae undergoes reduction while the other homologue is lost after failing to attach to the spindle on the metaphase plate (Orlando 1974). In the aphid species *Sitobion* near *fragariae,* Wilson & Sunnucks (1997) used molecular markers linked to sex chromosomes to demonstrate that the loss of one or the other X during the male formation is equally probable. In the spring, diapausing eggs hatch into fundatrices representing the first parthenogenetic generation. Since no recombination occurs during parthenogenesis (Blackman 1987), each resulting parthenogenetic genotype essentially represents a clone.

**Table 1.**
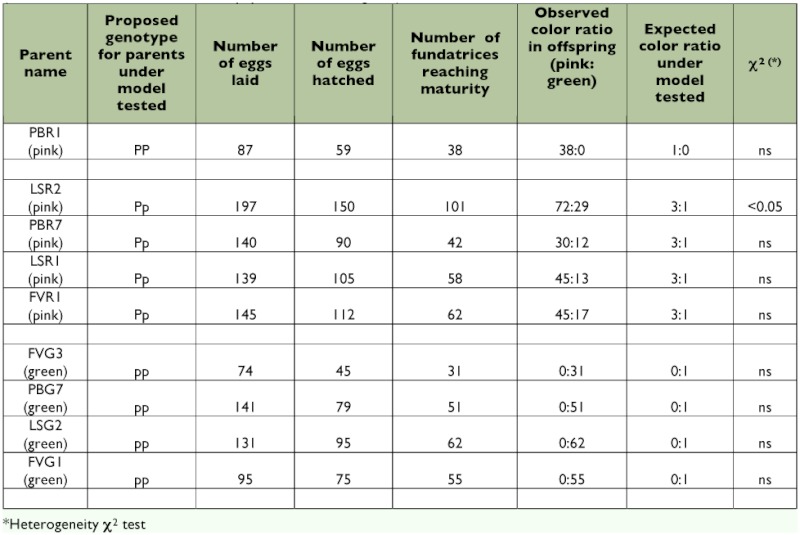
Pattern of colour morph production in selfed progeny generated by intraclonal matings in 5 pink genotypes and 4 green genotypes. The χ^2^ compares the observed colour ratio in the selfed offspring to the expected colour ratio under the genetic model tested (one autosomal locus, 2 alleles P and p, pink dominant to green).

Three experiments were designed to analyze the genetic basis of color polymorphism in pea aphids and test the simple genetic model — one locus and 2 alleles — proposed by Müller (1987). In experiment one, called “Intraclonal matings,” matings were performed for five pink clones and four green clones, and the ratio of pink versus green was evaluated in the resulting selfed offspring. Numbers of the two color morphs were then tested for departure from numbers expected under the assumption that one autosomal locus with two alleles is involved, using a heterogeneity χ2 test (Zar 1999) (Table 1). In experiment two, called “Male morph,” the color morph of males produced by five pink clones and four green clones was scored (Table 2) and the color ratio observed was compared to the expected pink:green ratios under two alternative models (the locus is autosomal or it is X-linked) using a heterogeneity χ^2^ test (Zar 1999). In experiment three, called “F1 and F2 Hybrid progeny”, 10 reciprocal crosses were performed between five pink clones and four green clones, considering pink*pink, green*green and pink*green matings (Table 3). The two putative genotypes for pink morphs were used (*Pp* and *PP*). Next, four reciprocal crosses were performed between eight randomly selected F1 hybrid clones (details about the origin of the F1 hybrids kept for producing the F2 generation are in Table 3) and generated eight F2 hybrid families (Table 4). The ratio of pink versus green color morphs obtained for each F1 and F2 offspring family was then compared to the expected ratio under the assumption of the involvement of a single biallelic locus located on the autosomes and not influenced by maternal effects, using a heterogeneity χ2 test (Zar 1999).

**Table 2.**
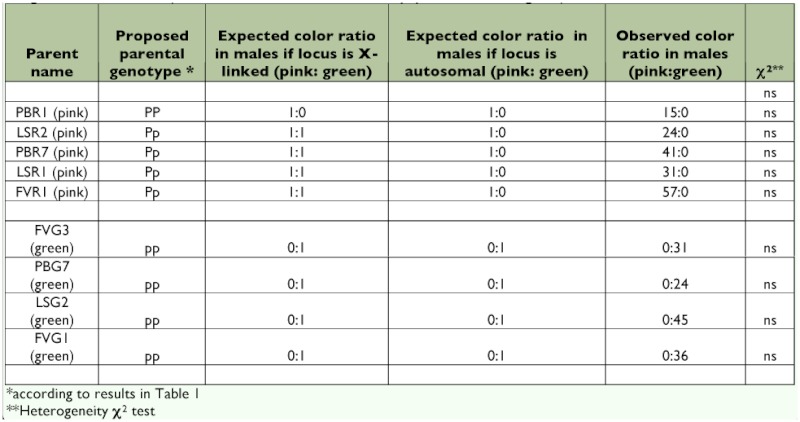
Color morph production in males (X0) for 5 pink sexuparae (XX) and 4 green sexuparae (XX). The χ^2^ compares the observed colour ratio in males produced by these sexuparae to the expected colour ratio in males under the genetic model tested (one autosomal locus, 2 alleles P and p, pink dominant to green).

The nine parental genotypes used for experiments one and two, and for crosses were collected in the summer of 1998 in fields located in the vicinity of Ithaca, NY, USA in an area of approximately 40 square miles and on the same plant species (M *sativa*, alfalfa). The genotypes were shown to be distinct using microsatellite loci (Caillaud et al., 2002, and unpublished). To induce the production of sexuals, five 3^rd^ instar parthenogenetic nymphs were taken from single genotype- stock cultures maintained at 20° C (16:8 L:D) and placed separately on alfalfa in a growth chamber at 18° C and a photoperiod of 13.5:10.5 L:D. The photoperiod was then decreased every three days by 15 minutes until it reached a photoperiod of 12.5:11.5 L:D (Caillaud et al. 2002). The temperature was then lowered to 16°C.

**Table 3.**
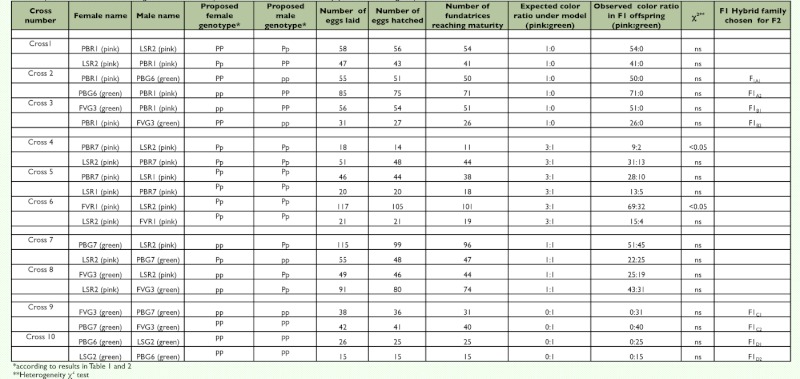
Colour morph production in F1 hybrids of 10 reciprocal crosses between 5 pink genotypes and 4 green genotypes. The χ^2^ compares the observed colour ratio in F1 hybrids to the expected colour ratio under the genetic model tested (one autosomal locus, 2 alleles P and p, pink dominant to green).

Starting six weeks after the beginning of the induction, sexual females and males were isolated from these stock cultures. In order to obtain virgin sexual females of known age, sexual females were isolated as nymphs from the stock culture and newly emerged adults were collected every day. Sexual females can be easily recognized by their thick hind tibia. Males can also be easily recognized by the presence of two black claspers close to the tip of the abdomen (Miyazaki 1987).

Crosses were performed as described in Via (1992). Three replicates of two males and three females for each direction of the cross were established. All fertilized eggs produced throughout the life of the females were harvested, surface sterilized and placed in an incubator under daily cycles of 4° C during a 10 hour day and 0° C during a 14 hour night. After about 100 days of this cold treatment, eggs were removed from the incubator and the hatchling progeny (fundatrices) were reared in Petri dishes containing alfalfa foliage until they reproduced. Color morphs were recorded on this 1^st^ parthenogenetic generation produced by fundatrices.

**Table 4.**
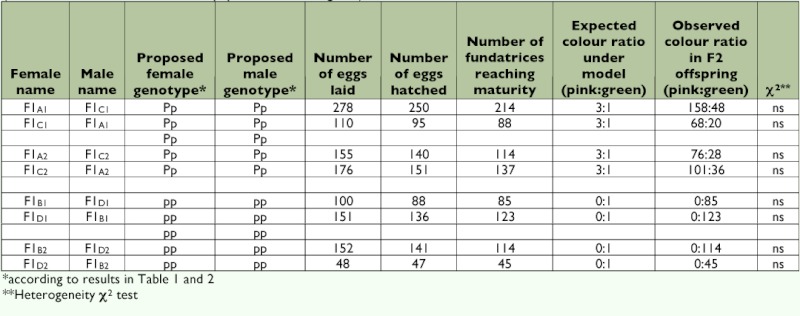
Color morph production in F2 hybrids of 4 reciprocal crosses between 8 F1 hybrids (see Table 3 for details on the origin of the F1 Hybrid parents). The χ^2^ compares the observed colour ratio in F2 hybrids to the expected colour ratio under the genetic model tested (one autosomal locus, 2 alleles P and p, pink dominant to green).

## Results

### Experiment 1: Intraclonal matings

Hatching success varied from 56.4% (PBG7) to 78.2% (FVG1). Between 46.6% (PBR7) and 68.8% (FVG3) of the fundatrices born survived. All fundatrices were dark green. Intraclonal mating in green clones (FVG3, for instance) produced 100% green offspring (ratio not significantly different from 0:1) (Table 1). In contrast, intraclonal mating in pink clones produced either 100%) pink offspring (case of PBR1) or pink and green individuals in a 3:1 ratio (case of all other pink clones). There was one exception. LSR2 intraclonal mating produced a pink:green ratio significantly different from 3:1 and equal to 2.5:1. Except for LSR2, observed results did not differ significantly from expected results under the assumption that one locus and two alleles (pink dominant, green recessive) are involved.

### Experiment 2: Male morph

If color polymorphism in pea aphid were influenced by loci on the X chromosome, pink sexuparae of heterozygous genotypes such as LSR2 or PBR7 (thus having the putative genotype X^P^X^p^) would produce 50% of pink males and 50% green males (recall here that XX sexuparae produce X0 males and that males can inherit either of the two X chromosomes). The pink versus green color ratio in males of these heterozygous clones would thus be not significantly different from 1:1. Table 2 shows that the four heterozygous pink genotypes studied (LSR2, PBR7, LSR1 and FVR1) produced 100% of pink males and their color ratio was not different from 1:0.

### Experiment 3: F1 and F2 hybrid progeny

Hatching success varied from 89.9% (F1_A1_ × F1_A1_) (Table 4) to 100% (LSR2 × FVR1) (Table 3). Between 85.6% (F1_A1_ × F1_A1_) (Table 4) and 100% (LSG2 × PBG6) (Table 3) of the fundatrices born survived. All fundatrices were dark green. In F1 hybrids as well as in F2 hybrids, the direction of the cross did not affect the ratio of pink versus green individuals produced (Tables 3, 4). Mating between green clones, either parental clones, or F1 hybrid clones always produced 100% green offspring. In contrast, as already recorded in Table 1 for intraclonal matings, mating between pink forms produced either 100% pink individuals or pink and green individuals in a 3:1 ratio. There were two departures from the 3:1 ratio. Mating between females of PBR7 and males of LSR2 produced pink and green individuals in a 4.5:1 ratio while mating between females of FVR1 and males of LSR2 produced pink and green individuals in a 2.1:1 ratio (Table 3). Except for these two exceptions, observed results did not differ significantly from expected results under the assumption that one autosomal locus and two alleles (pink dominant, green recessive) are involved.

## Discussion

The results of this study are most simply explained if color morph is determined by alternative alleles at a single autosomal locus (pink being dominant to green), without influence of cytoplasmic factors and/or maternally inherited symbionts. Specifically, no significant departure of the color ratio observed in selfed and hybrid progeny from the expected ratio under the assumption of a biallelic locus was found (Table 1, 3, and 4). In addition, the color ratio observed for hybrid progeny did not vary with the direction of the cross (Tables 3, 4). Last, pink heterozygous (*Pp*) sexuparae gave birth to 100% of pink males (Table 2). It is worth noting that this locus, hereafter called *colorama*, appears to be limited in expression to some forms since fundatrices hatched from eggs do not show phenotypic variation for color morphology (they are always dark green), regardless of their *colorama* genotype. Also, segregation ratios were observed that differed from the expected ratios when LSR2 males, but not LSR2 females, were involved. Since males and females of LSR2 were genetically identical except for the number of sex chromosomes (males have one copy of each X-linked gene while females have two copies), this would suggest that there is an interaction between the *colorama* locus and genes on the X chromosome.

Based on previous studies of color determination, it seems plausible that the *colorama* locus is linked to phenotypic expression of color through a relatively simple mechanism. The biochemical genetics of pigmentation is best characterized in mammals, in the laboratory mouse in particular. Two genes, the melanocortin-1-receptor (*MCR1*), a G-protein coupled receptor highly expressed in pigment-producing cells, and the agouti-signaling gene (*Agouti*), an antagonist of *MCR1,* control in large part the relative production of black pigments and yellow/red pigments in mice (Barsch 1996). In aphids, variation in ground color depends on the type and/or relative amounts of two types of pigments in the haemolymph, aphins, and carotenoids (Jenkins et al. 1999). For example, the tulip tree aphid, *Macrosiphum liriodendra*, has green and pink genotypes depending on the relative amounts of different carotenoid pigments present (Weisgraber et al. 1971). Also, in *S. avenae,* a brown and a green clone were shown to differ by the type of carotenoids present (four carotenes for the brown clone against one form for the green clone) and the amount of carotenoid (the brown clone had three times more carotenoid material than the green clone) (Jenkins et al. 1999). It is thus tempting to hypothesize that *colorama* could play a role in the biosynthesis of carotenoids in pea aphids.

The *colorama* locus could also be involved in the color polyphenism known in pea aphids. Environmentally-induced variation in body color has long been described in aphids. For instance, in *Aphis gossypii*, a single clone was described as greenish-black on cotton and as light yellow on broad bean, showing that variation in color can be induced by a change in the diet (Watt and Hales 1996). In *A. pisum,* Fröhlich (1962) reported that clones that were pink and green became yellow if kept at temperatures of 30° C to 35° C. A more recent study shows that some pink clones become yellow if kept at 15° C, while green clones never become yellow at such temperature (John Losey, unpublished). Although maternally-inherited bacterial symbionts which occur in specialized structures in the aphid abdomen could be associated with such changes (Houk 1974; Brown 1975; Jenkins et al. 1999), color genes could play a role too. It is suspected that polymorphisms and polyphenisms share similar genetic and developmental architectures (Braendle et al. 2004). In many instances a similar alternative phenotype is expressed as an environmentally controlled polyphenism in some species and as a genetically controlled polymorphism in another (closely related) species (Nijhout 1999). Also, a similar phenotype can be determined by either the environment or variation at a single locus. In the buckeye butterfly, *Precis coenia,* the background surface coloration of the ventral hind wing varies between the autumn morph “rosa” and the summer morph “linea”. The autumn morph is usually induced by low temperature and short days, but there is also a gene (*rosa*) whose recessive allele produces the autumn phenotype when homozygous (Rountree and Nijhout 1995). It appears likely that there is a physiological/functional link between polyphenisms and polymorphisms. In pea aphids, both a color polyphenism and a color polymorphism coexist in a single organism, and a single genotype, which may help in the study of the interplay between polyphenism and polymorphism.

Establishing the genetic basis for color polymorphism will greatly facilitate prediction of the response of pea aphid populations to selection pressures that operate differentially on the two color morphs. Losey et al. (1997) provide a model that predicts relative densities of pink and green aphid morphs under differing selective pressures from predators (that prey more heavily on pink morphs) and parasitoids (that parasitize a higher proportion of green morphs). This model is valid for the summer months when mating does not occur and thus densities of green and pink morphs vary independently almost as two sympatric populations. With the establishment of the genetic basis for pea aphid color polymorphism, this model can be improved to account for mating and subsequent genetic dominance of the pink allele over the green one. Moreover, this model could be generalized for other differential selective pressures (such as “bottom up” pressure from the host plant) and permit longer-term evolutionary hypotheses to be generated and tested.

Color morph variation is a model system for establishing a link between genotypes and ecologically relevant phenotypes (Brakefield 1998; Eizirich et al. 2003; Nachman et al. 2003). Taking advantage of the fact that a large number of mammalian pigmentation genes have been characterized at the molecular level, Nachman et al. (2003), working on the non-model organism rock pocket mice *Chaetodipus intermedius*, have been able to analyze particular mutations in the well characterized pigmentation gene *MC1R* in various lava-dwelling mice populations exhibiting adaptive melanism, and to look for associations between those mutations and the adaptive dark color. The existence of a simple genetic determinism for color polymorphism in pea aphids, a system in which genetic investigation is possible (Hawthorne and Via 2001; Braendle et al. 2005a, b; International Aphid Genomics Consortium, 2010) may allow investigations linking genetics (alleles at *colorama*), biochemistry (type and amount of carotenoid), ecology (fitness of different color morphs) and evolutionary biology (mechanisms that maintain genetic variation for color genes).
